# Varicella: epidemiological aspects and vaccination coverage in the Veneto Region

**DOI:** 10.1186/1471-2334-9-150

**Published:** 2009-09-08

**Authors:** Vincenzo Baldo, Tatjana Baldovin, Francesca Russo, Marta Cecilia Busana, Cinzia Piovesan, Greta Bordignon, Aurore Giliberti, Renzo Trivello

**Affiliations:** 1 Department of Environmental Medicine and Public Health, Institute of Hygiene, University of Padua, Padua, Italy; 2 Regional Department for Prevention, Public Health and Screening Section, Veneto Region, Italy

## Abstract

**Background:**

With the control of many infections through national vaccination programmes, varicella is currently the most widespread preventable childhood disease in industrialized nations. In 2005 varicella vaccination was added to the Veneto Region routine immunization schedule for all children at 14 months of age and 12 year-old susceptible adolescents through an active and a free of charge offer. To evaluate parameters at the start of the programme, we conducted a study to describe the epidemiology of varicella infection and coverage rates for varicella vaccine in the Veneto Region (North-East Italy).

**Methods:**

We examined incidence rates and median age of case patients in the Veneto Region for 2000-2007 period using two data sources: the mandatory notification of infections diseases and the Italian Paediatric Sentinel Surveillance System of Vaccine Preventable Diseases. Corrected coverage rates were calculated from data supplied by the Public Health and Screening Section of the Regional Department for Prevention.

**Results:**

In the Veneto Region from 2000 to 2007, a total of 99,351 varicella cases were reported through mandatory notifications, mostly in children under 15 years of age. The overall standardised annual incidence ranged from 2.0 to 3.3 per 1,000 population, with fluctuations from year to year. The analysis by geographic area showed a similar monthly incidence rate in Italy and in the Veneto Region. The vaccination average adherence rate was 8.2% in 2004 cohort, 63.5% in 2005 cohort and 86.5% in 2006 cohort. Corrected coverage rates were 8.1% in 2004 cohort, 59.9% in 2005 cohort and 70.0% in 2006 cohort, respectively.

**Conclusion:**

Data from passive and active surveillance systems confirm that varicella is a common disease which each year affects a large proportion of the population, mainly children. Uptake of the varicella vaccination programme was strikingly good with average coverage rates of about 70% after only 3 years. Sustained implementation of existing vaccine policies is needed to warrant any significant reduction of varicella incidence in the Veneto Region. Continued surveillance will be important to monitor the impact of the recently introduced mass vaccination policy.

## Background

Varicella-zoster virus is the causal agent of varicella (chickenpox) currently the most widespread preventable childhood disease in industrialised nations where many other infections are well-controlled by national vaccination programmes [1,2]. Primary varicella infection is a highly contagious illness characterised by a maculo-papulo-vesicular rash associated with fever and malaise [3]. It is usually considered a mild self-limiting disease. Nevertheless, serious complications such as bacterial super-infection, pneumonia, central nervous system diseases can occur and may lead to hospitalisation and even death [2,4]. Infants, adolescents and immunocompromised people are at higher risk [5].

In a non-vaccinated population the risk of acquiring varicella is over 95% and most people contract this infection before the age of 20 [2]. The disease is endemic in most populations worldwide. According to the Centres for Disease Control and Prevention (CDC), the incidence of varicella is assumed to approximate the annual birth cohort [6].

In the absence of universal childhood vaccination programme, varicella incidence peaks in children under 10 years of age, with the highest incidence rates between 3 and 6 years of age [7].

Prevention of infection by vaccination is the optimal approach in the management of varicella. World Health Organization recommends routine childhood immunization to be considered in countries where the disease is a relatively important public health and socio-economic problem and where high and sustained vaccination coverage rates can be achieved [8]. Indeed, in case of suboptimal coverage rates, the positive effects of vaccinating children might be offset by a shift of infection to older age-groups who are at risk of more severe disease [2,9]. After the licensure of varicella vaccine in the United States (US) in 1995, the Advisory Committee on Immunisation Practices (ACIP) recommended routine vaccination with one dose of varicella vaccine for all children 12-18 months of age and catch up of all susceptible children 19 months to 12 years of age; vaccination with 2 doses was recommended for all susceptible people ≥ 13 years of age [5]. The implementation of these recommendations was followed by a marked decline in the incidence of varicella, its complications and related mortality [1,10-12]. However, while varicella incidence reached its lowest level, further decrease was not observed and outbreaks in school populations with high coverage rates for varicella vaccination were reported [13-17]. To further reduce varicella incidence and its complications and to control outbreaks, ACIP issued new recommendations on the use of varicella vaccine including a second dose routinely administered at 4 to 6 years of age in those subjects previously vaccinated with 1 dose [5].

In January 2005, varicella vaccine was introduced in the Veneto Region (North-East Italy) vaccination schedule (Regional Law No. 4403 of 30 December 2005). The active and free of charge offer concerned children aged 14 months and 12 years-old adolescents with a negative history for varicella. Following ACIP recent discussion on varicella vaccination, the Veneto Region expanded its recommendation to a second dose of varicella vaccine for 6 year-old children as part of routine childhood immunisation and a catch up dose for teenagers was confirmed. In this paper we discuss the epidemiological trends of varicella infections as well as varicella vaccination coverage rates in the Veneto Region.

## Methods

Epidemiological data included all new cases of varicella reported, in the period 2000-2007, to the Regional Department for Prevention, Public Health and Screening Section, in accordance with the Italian Communicable Disease Act. For each subject affected by chickenpox, available data include specific information on the date of diagnosis, varicella vaccine status and demographic data.

To conduct a more in-depth epidemiological evaluation, we also analysed incidence data from a nationwide sentinel system, the Italian Paediatric Sentinel Surveillance System of Vaccine Preventable Disease (SPES). Data are available from the official web-site coordinated by the National Institute of Health [18].

Furthermore, with the launch of varicella vaccination programme on 1^st ^January 2005, we studied vaccination coverage rates observed in 14 months-old children. A retrospective analysis was conducted from 1^st ^January 2005 to 31^st ^December 2007 considering all infants born between 2004-2006 in the Veneto Region.

At the start of the vaccination programme, only monovalent varicella vaccine were distributed in Italy, Varivax^® ^(manufactured by Merck and distributed in Europe by Sanofi Pasteur MSD) and Varilrix^® ^(Glaxo Smith Kline). In the spring of 2007, a quadrivalent combination measles, mumps, rubella, and varicella (MMRV) vaccine (ProQuad^® ^manufactured by Merck and distributed in Europe by Sanofi Pasteur MSD) was made available as an alternative for concomitant vaccination against the 4 diseases.

Data was supplied by the Public Health and Screening Section of the Regional Department for Prevention. Public Health Services that includes information obtained from all of the 21 Veneto Health Care Units. Data included the number of eligible children in the investigated period (resident population of children born between 1/1/2004 and 12/31/2006) and the number of 14 months-old children vaccinated against measles, mumps, rubella and varicella.

Annual and age specific incidence rates were calculated per 1,000 population, using the latest census data for year 2001 (National Institute of Statistics) and data provided by the Veneto Region Statistical Bureau for the remaining years.

Children with a reliable history of varicella as well as those previously vaccinated were deemed to be protected [6]. Corrected coverage rate for varicella was calculated to estimate the proportion of children protected against varicella: both children who received the first dose of MMRV quadrivalent combination or monovalent varicella vaccine as well as those with a positive history of varicella infection were considered for this calculation.

Furthermore, adherence rates were calculated on the population responding to the active offer (solicited children) taking into account the total number of vaccinated children (with MMRV or varicella vaccine). The population of solicited children comprised all the children who received measles vaccine (all formulation types) and excluded subjects with a history of varicella infection.

## Results

In the Veneto Region, from 2000 to 2007, a total of 99,351 varicella cases were reported through mandatory notifications of infections diseases. During this period, the overall standardised annual incidence ranged from 2.0 to 3.3 per 1,000 population, with fluctuations from year to year. Most of the cases were reported during the spring months with an incidence rate peaking in children aged 1 to 4 years (31.3 cases per 1,000 population). Ninety one percent of cases occurred among children under 15. Adults had the lowest rate of reported cases and accounted for 8.0% (0.3 per 1,000 population), as shown in Table 1.

**Table 1:** Reported cases of varicella cases and rates per 1,000 population among the Veneto Region residents by age group and year.

Year	Age group													
	
	<1		1-4		5-9		10-14		15-19		≥ 20		Total	
**2000**	207	(4,8)	5256	(30,6)	5215	(25,5)	949	(4,6)	316	(1,6)	1182	(0,3)	13188	(2,9)
**2001**	179	(4,3)	5094	(30,1)	4561	(22,6)	693	(3,5)	201	(1,0)	1047	(0,3)	11871	(2,6)
**2002**	209	(4,8)	5139	(30,0)	4775	(23,4)	674	(3,3)	202	(1,0)	952	(0,3)	11985	(2,6)
**2003**	243	(5,5)	6085	(34,8)	5302	(25,4)	730	(3,5)	203	(1,0)	1133	(0,3)	13753	(3,0)
**2004**	291	(6,3)	6926	(39,0)	5871	(27,4)	762	(3,6)	208	(1,0)	1225	(0,3)	15283	(3,3)
**2005**	217	(4,7)	4611	(25,5)	3505	(15,9)	377	(1,8)	150	(0,7)	944	(0,2)	9819	(2,1)
**2006**	279	(6,0)	6685	(36,4)	4982	(22,3)	615	(2,9)	163	(0,8)	895	(0,2)	13644	(2,9)
**2007**	181	(3,8)	4511	(24,1)	3956	(17,4)	428	(2,0)	98	(0,4)	632	(0,2)	9808	(2,0)

**Figure 1:**
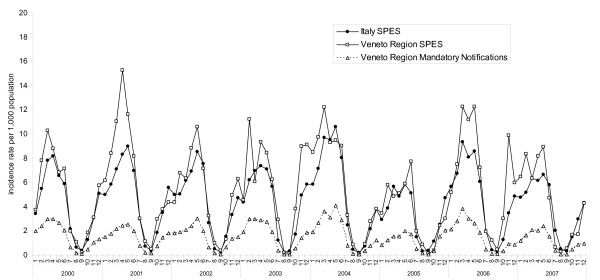
**Monthly incidence rate in children 0-14 years old per 1,000 population reported by SPES and mandatory notifications (2000-2007)**.

SPES data on varicella showed incidence rates per 1,000 population in children less than 14 years of 53.4 in 2000, 57.4 in 2001, 54.6 in 2003, 66.6 in 2004, 40.5 in 2005, 60.6 in 2006 and 46.3 in 2007, respectively (Figure 1). Similar monthly incidence rates were reported for Italy and the Veneto Region, with a mean ratio of 0.9 (range 0.3 to 3.2). Data obtained from the different surveillance systems for the Veneto Region showed monthly varicella incidence in 0-14 years-old children ranging from 0.1 to 15.3 per 1,000 population as reported by SPES, while data obtained through mandatory notifications ranged from 0.0 to 4.3 per 1,000 population. Overall, sentinel network incidence data was 4.2 time higher than that reported by statutory notification (range 1.1-11.9).

A summary of vaccinated children in the target population by cohort of birth is given in Table 2. The average adherence rate was 8.2% in 2004 cohort, 63.5% in 2005 cohort and 86.5% in 2006 cohort. Corrected coverage rates were 8.1% (range 2.6 - 18.5%) in 2004 cohort, 59.9% (range 27.7 - 79.9%) in 2005 cohort and 70.0% (range 51.5- 85.2%) in 2006 cohort. MMRV and monovalent varicella vaccine were administered to 2005 cohort in 2.9% (1,312) and 55.1% (25,320) children repectively, while the same vaccines were almost equally gave to 2006 cohort.

**Table 2:** Number of vaccinated children and coverage rate by birth cohort.

	Birth cohort		
	
	2004	2005	2006
**Resident children**	45934	45918	45047
**Susceptible children**	45303	45064	44407
**Solicited children**	37717	41935	35477
**Adherence rate (%)**	8.2	63.5	86.5
**Vaccinated children**	3103	26632	30692
measles, mumps, rubella, and varicella vaccine	44	1312	15606
monovalent varicella vaccine	3059	25320	15086
**Coverage rate [range] (%)**	6.8 [0.9-18.3]	58.0 [24.6-79.1]	68.1 [48.6-82.7]
**Corrected coverage rate [range] (%)**	8.1 [2.6-18.5]	59.9 [27.7-79.9]	69.7 [51.5-85.2]

## Discussion and conclusion

In the Veneto Region the epidemiology of varicella shows a seasonal pattern with a peak incidence during the spring months (March thought May), and a lower incidence in late summer and early autumn. Few studies in the literature analysed seasonal trends of varicella infections. Indeed, most papers focus on the complications related to varicella such as hospitalisations and deaths [5,11,19]. Our data confirm the burden of varicella, a common disease which affects a large proportion of the population each year, as demonstrated by the high overall standardised annual incidence rate. As expected, the infection is predominantly a paediatric disease with only 9.6% of cases in subjects ≥ 15 years; these results are in line with recent Italian seroprevalence data [20]. Although it is too early to observe an effect of the new vaccination schedule, such baseline data are precious for further evaluation of the programme, in particular with regard to its impact on the incidence of varicella and its complications over time and possible herd immunity effects. In the US, active surveillance conducted in three different areas showed a dramatic decrease in disease incidence, correlated hospitalizations and mortality after only 5 years of universal vaccination programme. With vaccination coverage rates in 19-35 months-old children ranging from 74-84%, varicella cases were reduced by 71-84% [1] while corresponding data after 11 years of varicella vaccination, were 92-93% for coverage rates and ~90% for varicella incidence decline as observed in two different surveillance areas (89.9% and 90.4%) [21].

In our paper we examined data from the national routine notification system. This is a passive surveillance system which is useful to analyse trends of varicella but inaccurate to measure disease incidence due to under notification and under diagnosis [22]. Indeed, data observed by SPES, a voluntary paediatric sentinel surveillance system, were 1.1 to 11.9 times higher than those obtained through mandatory notifications, clearly showing a substantially higher sensitivity of SPES sentinel surveillance system when compared to compulsory notifications, as already shown in previous studies [20,23].

Overall, varicella has a significant impact that can be addressed by universal vaccination much better than symptomatic and antiviral treatments [3]. Prevention has a number of overall health care advantages, in particular with regard to reduction of complications and health care costs as well as improvement in quality of life [11,12,24].

In this scenario the Veneto Region in 2005 introduced an universal vaccination program for varicella, aiming at achieving high vaccination coverage in the target population in order to prevent the accumulation of a susceptible population at risk of more severe disease. Hence it is fundamental to reach high coverage rates in the shortest time [25]. The rapid uptake of varicella vaccination in the Veneto Region is striking, reaching in just three years a corrected coverage rate of 69.7%, very close to target vaccination coverage of 80% for children 14 month-old. Of note, to calculate corrected coverage rates we estimated the proportion of varicella history positive subjects using mandatory notifications. While these latter represented 2% in our study, it is interesting to mention that another recent study conducted in the same area reported 7% subjects with varicella history [26]. Overall, our study might have underestimated the true corrected coverage rates.

According to a model developed by the Italian National Institute of Health and Tor Vergata University (Rome), a vaccination coverage of 80% for newborns (14 months) and 50% in susceptible adolescents (12 years-old) would result in a 94% drop in varicella cases [27]. Continued monitoring will be needed to understand the impact of vaccination on the epidemiology of this disease and its complications, including herpes zoster [3,28,29].

Similar to ACIP recommendations in 2007 [5], our programme introduced the use of a combined multivalent MMRV vaccine as an alternative to measles, mumps, rubella and varicella monovalent vaccine in separate injections, offering the convenience of a single injection to facilitate the introduction of varicella vaccination within the routine childhood immunisation schedule [30]. However, US surveillance systems detected a signal of increased incidence of febrile seizures in subjects vaccinated with MMRV when compared to separate vaccines, particularly after the first dose. Consequently, MMRV was withdrawn by ACIP in February 2008 without any change in overall recommendations for varicella vaccination. A workgroup was also nominated to continue the monitoring of MMRV safety and provide relevant data for future policy options [31]. In Veneto, the choice of MMRV over separate injections is left to the physician and subject decision while monitoring of safety is already in place through a very efficient pharmacovigilance system which represents an invaluable tool to assess vaccine safety in the long term.

Universal vaccination can dramatically reduce the incidence of varicella, varicella associated complications, hospitalisation rates and fatality and this has been demonstrated through the large US experience [21]. Sustained implementation of existing vaccine policies is needed to warrant any significant reduction of varicella incidence in the Veneto Region. Additionally, a parallel surveillance of the disease in the population and coverage rates among adolescence as well as young children will be fundamental to evaluate the impact of the vaccination strategy and policy.

## Competing interests

The authors declare that they have no competing interests.

## Authors' contributions

VB participated to conceive and design the study, to collect and analyse data, and in the overall coordination and drafting of the manuscript; TB and MCB participated in the study conception and design, in data analysis and collection, and in the write-up; FR participated in conceiving and designing the study; CP participated in the study acquisition and coordination of data collection; GB and AG participated in the data analysis and collection; RT reviewed the study design and manuscript. All authors have read and approved the final manuscript.

## Pre-publication history

The pre-publication history for this paper can be accessed here:

http://www.biomedcentral.com/1471-2334/9/150/prepub
